# Comparison in Terms of Accuracy between DLP and LCD Printing Technology for Dental Model Printing

**DOI:** 10.3390/dj10100181

**Published:** 2022-09-28

**Authors:** Ioannis A. Tsolakis, William Papaioannou, Erofili Papadopoulou, Maria Dalampira, Apostolos I. Tsolakis

**Affiliations:** 1 Department of Orthodontics, School of Dentistry, Aristotle University of Thessaloniki, 541 24 Thessaloniki, Greece; 2 Department of Preventive & Community Dentistry, School of Dentistry, National and Kapodistrian University of Athens, 157 72 Athens, Greece; 3 Department of Oral Medicine & Pathology and Hospital Dentistry, School of Dentistry, National and Kapodis-Trian University of Athens, 106 79 Athens, Greece; 4 Private Practice, 541 24 Thessaloniki, Greece; 5 Department of Orthodontics, School of Dentistry, National and Kapodistrian University of Athens, 157 72 Athens, Greece; 6 Department of Orthodontics, Case Western Reserve University, Cleveland, OH 44106, USA

**Keywords:** 3D printing, accuracy, dental models, orthodontics, dentistry

## Abstract

Background: The aim of this study is to evaluate the accuracy of a Liquid Crystal Display (LCD) 3D printer compared to a Direct Light Processing (DLP) 3D printer for dental model printing. Methods: Two different printers in terms of 3D printing technology were used in this study. One was a DLP 3D printer and one an LCD 3D printer. The accuracy of the printers was evaluated in terms of trueness and precision. Ten STL reference files were used for this study. For trueness, each STL file was printed once with each 3D printer. For precision, one randomly chosen STL file was printed 10 times with each 3D printer. Afterward, the models were scanned with a model scanner, and reverse engineering software was used for the STL comparisons. Results: In terms of trueness, the comparison between the LCD 3D printer and DLP 3D printer was statistically significant, with a *p*-value = 0.004. For precision, the comparison between the LCD 3D printer and the DLP 3D printer was statistically significant, with a *p*-value = 0.011. Conclusions: The DLP 3D printer is more accurate in terms of dental model printing than the LCD 3D printer. However, both DLP and LCD printers can accurately be used to print dental models for the fabrication of orthodontic appliances.

## 1. Introduction

Three-dimensional printing technology has been a research area of interest from different aspects in dentistry and more specifically orthodontics. Initially, three-dimensional printing technology was used in orthodontics to fabricate dental casts. Those dental casts were used for diagnosis and treatment purposes. The first application in terms of treatment was in clear aligner fabrication and later the fabrication of different customized appliances or for the fabrication of sterilizable aerosol aspirators. The evolution of scanners and the creation of intraoral scanners gave clinicians the ability to take impressions without causing any discomfort to the patients. Those two technologies gave the ability to doctors to have a complete digital workflow in everyday practice [1,2,3,4,5,6,7,8].

The first introduction of three-dimensional printing technology was in 1986 by Charles Hull. Charles Hull introduced Stereolithography (SLA), the first type of three-dimensional printing technology. Four years later, the fused deposition modeling (FDM) three-dimensional printing technology was introduced by Scott Crump. Currently, there are many different types of three-dimensional printing technologies. The most commonly used 3D technologies in dentistry are the SLA, Direct Light Processing (DLP), and Liquid Crystal Display (LCD). All these technologies are based on a vat polymerization technique. These printers use a liquid photopolymer resin, curing it with the usage of a light source [9,10,11,12,13,14,15,16,17,18,19].

It is proven that DLP technology delivers highly accurate results, which is why it is widely used in dentistry and more specifically in orthodontics. Another advantage of DLP technology is that it can provide 3D printed models in a much shorter time than SLA 3D printers [9]. DLP technology is based on a digital light projector which is the source of light to photo-polymerize the resin. This projector flashes an image of a layer at once and this makes the procedure much faster because all points of a layer are cured at the same time. A DLP printer is characterized by an LED screen which is composed of a digital micromirror device (DMD). These small micromirrors are able to concentrate the light and form the structure of a layer on the bottom of the resin tank. At the end of the printing process such as SLA, the model needs to be washed and post-cured [9].

LCD printing technology is becoming very popular because it is more affordable than the other vat polymerization 3D printers. They are more affordable because the parts of an LCD 3D printer and more specifically the light source have a lower cost than other 3D printers [9]. A Liquid Crystal Display is used as a light source for these printers. This Liquid Crystal Display is composed of LCD panels that allow the light to shine in parallel and come through onto the build area. In this 3D printing technology, there is no need to expand the light through lenses or other devices. This process gives the advantage to this 3D printing technology because the result cannot be affected by any pixel distortion. According to the available literature, there are not many studies that evaluated the accuracy of LCD 3D printing technology [9].

The accuracy of any medical device and more specifically any 3D printer should be evaluated before any clinical application. According to the literature, the best way to evaluate the accuracy of a 3D printer is to study the precision and the trueness of its results. Precision is the part of accuracy that studies the evaluation of the results compared to each other that resulted from the same 3D file. In other words, higher precision means that the machine can give more repeatable and consistent prints. Trueness is the part of accuracy which measures the deviation of each result when compared to the actual object dimensions. More specifically, high trueness means that the machine can give results that are close to or equal to the actual dimensions of the digital 3D object [20,21,22,23,24,25,26,27,28,29,30,31,32,33,34].

The aim of this study is to evaluate the accuracy in terms of trueness and precision of a Liquid Crystal Display (LCD) 3D printer compared to a Direct Light Processing (DLP) 3D printer for dental model printing.

## 2. Materials and Methods

Maxillary and mandibular arches in a Standard Triangle Language (STL) form resulting from intraoral scans were used in this study. All files were recruited from private practice. All arches were well aligned in order to follow the inclusion criteria and standardize the methodology of this study. The selected arches should not appear with any other appliances on them or any aligner attachments. A power analysis was conducted in order to calculate the power significance of the results based on a previous study [31]. The power analysis suggested that a minimum sample size of 10 subjects will give a confidence level of 95%. Thus, 10 subjects (5 maxillary and 5 mandibular dental arches) were decided to be selected for this study.

**Figure 1:**
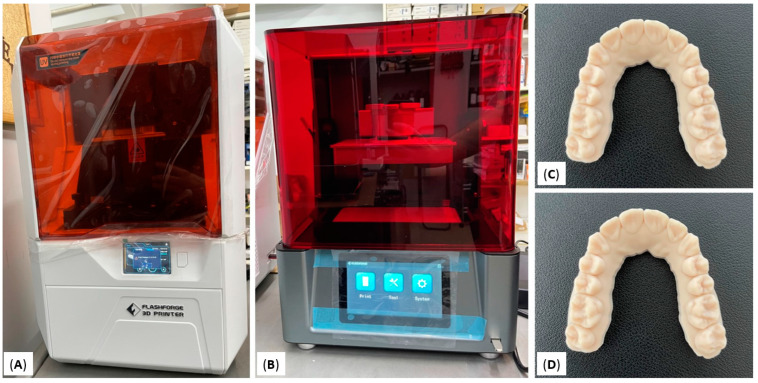
(**A**) LCD 3D printer, Flashforge Focus 9.25 6K; (**B**) DLP 3D printer, Flashforge Hunter; (**C**) printed model with Flashforge Focus 9.25 6K; (**D**) printed model with Flashforge Hunter.

The STL files were recruited from well-aligned dental arches that were scanned with an intraoral scanner (CS 3700, Carestream Dental LLC, Atlanta, GA, USA). All STL files coming from the same intraoral scanner were used as reference models. Afterward, these files were printed using an LCD and a DLP 3D printer. The LCD printer of choice was Flashforge Focus 9.25 6K (Zhejiang Flashforge 3D Technology Co., Ltd., Jinhua, China) and the DLP printer of choice was Flashforge Hunter (Zhejiang Flashforge 3D Technology Co., Ltd., Jinhua, China) (Figure 1). For the consistency of the study, a third-party resin was used for both LCD and DLP technologies (U-ORTHO almond HD, Unishape, Thessaloniki, Greece). The layer of thickness for all prints was set at 100μm. All models were printed in horizontal position on the building template. Once the print was completed the models were rinsed with isopropyl alcohol (IPA) and cured with a UV lamp.

**Figure 2:**
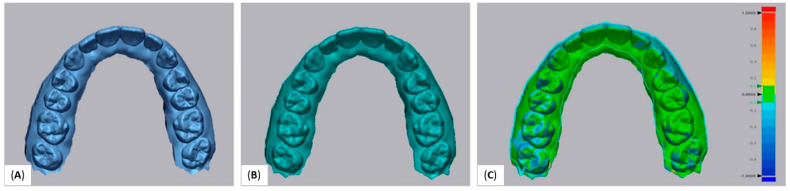
(**A**) Reference STL file, (**B**) STL file resulted from the 3D printed model, (**C**) 3D comparison of the two STL files.

All 10 STL files were printed once with each printer in order to evaluate the trueness of the two printers. For the consistency of the study, each printing template contained 1 dental cast in a horizontal position. This is because the template of the DLP 3D printer was much smaller (120 × 67.5 × 150 mm) than the LCD 3D printer (197 × 122 × 200 mm). Once the printing of models was completed, each model was scanned with a model scanner (D900, 3Shape, Copenhagen, Denmark). We performed the scanning immediately after the printing to avoid distortion of the models [35,36]. Subsequently, all scanned STL files of 3D printed models were compared with the corresponding reference STL files. In order to evaluate the precision of 3D printers, one randomly selected STL file was printed 10 times with both 3D printers. All models were scanned with the model scanner (D900, 3Shape, Copenhagen, Denmark) when the printing of models was completed. This scanner consists of 4 cameras of 5.0 MP and its accuracy is proven to be as low as 6 μm [37]. Afterward, all scanned STL files of 3D printed models were compared with the reference STL files. For the evaluation of trueness and precision, a reverse engineering software Geomagic Control X (version 2020.1; 3D Systems, Inc, Rock Hill, SC, USA) was used to compare the STL files. The best-fit three-dimensional superimposition method was used. According to this method, best-fit mathematic algorithms are used to overlay the STL file to the reference one. The variance across the experimental file in comparison with the reference cast file resulted from the superimposition of the STL files. The value used to compare the files is the root mean square value (RMS) which corresponds to the differences between the reference STL file and the scanned STL file (Figure 2).

### Statistical Analysis

All measurements were filed in an Excel spreadsheet (Microsoft, Redmond, WA, USA). For the statistical analysis, SPSS software (version 27; IBM, Armonk, NY, USA) was used. Shapiro–Wilk test was used to examine the distribution of the data. The distribution of the RMS measurement by printer type is described in relevant tables and figures. Differences in RMS values between printers were tested using a paired *t*-test test for both trueness and precision measurements. All tests are 2-sided at a 5% level of statistical significance.

## 3. Results

The total sample of this study was 10 dental arches, 5 maxillary and 5 mandibular dental arches. Intraclass correlation was performed on five randomly selected subjects in order to test the operator’s reliability with the Geomagic Control X software. The measurements took place once by superimposing the STL files, and three weeks after all measurements were repeated. The ICC tests showed no differences between the two time measurements, with an excellent correlation which ranged from 0.988 to 0.996 for intra-observer reliability, meaning the operator could not affect the software calculations. The printing time per model for the LCD printer was 32 min and for the DLP printer was 35 min.

### 3.1. Trueness

**Figure 3:**
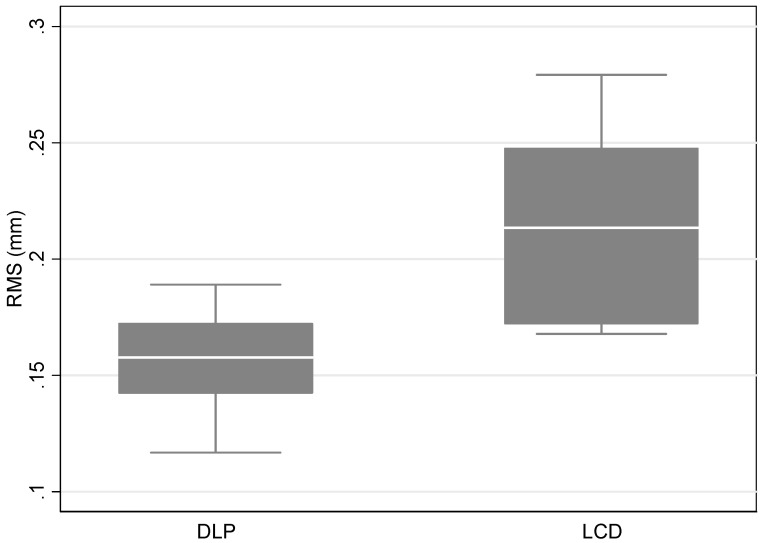
Boxplot of the distribution of RMS measurements by printer type.

According to the measurements of trueness, the mean RMS value for the LCD 3D printer Flashforge Focus 9.25 6K was 0.21 mm ± 0.04 and for the DLP 3D printer Flashforge Hunter was 0.16 mm ± 0.02 mm. Overall, there were significant differences between the two printers with a *p*-value < 0.05. More specifically, the comparison between the Flashforge LCD 3D printer and Flashforge DLP 3D printer was statistically significant with a *p*-value = 0.004 (Table 1, Figure 3).

### 3.2. Precision

**Figure 4:**
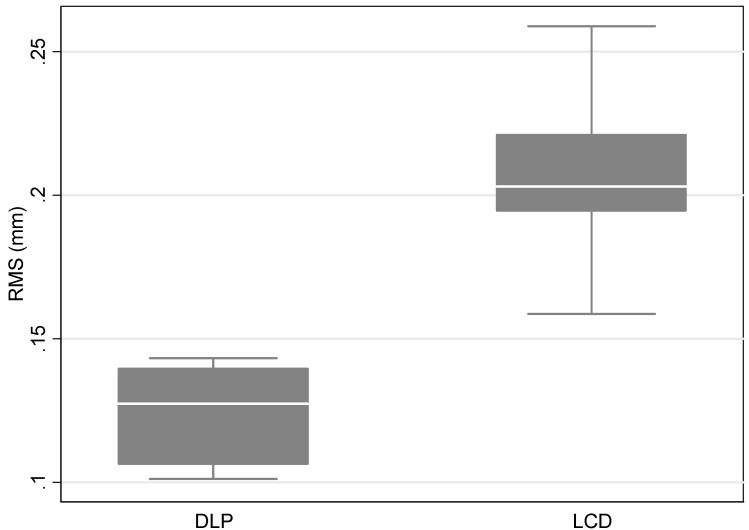
Boxplot of the distribution of RMS measurements by printer type.

According to the measurements of precision, the mean RMS value for Flashforge Focus 9.25 6K was 0.21 ± 0.04 mm, and for the DLP 3D printer Flashforge Hunter was 0.12 ± 0.02 mm. Overall, there were significant differences between the two printers, with a *p*-value < 0.05. More specifically, the comparison between the Flashforge LCD 3D printer and Flashforge DLD 3D printer was statistically significant, with a *p*-value = 0.011 (Table 2, Figure 4).

## 4. Discussion

This study compared two different 3D printing technologies in terms of accuracy for printing dental models. In order to evaluate the accuracy of each printer, we investigated the trueness and precision of each machine.

According to the present literature, there are plenty of studies that examined the accuracy of DLP 3D printing technology, while there are not many studies that evaluated the accuracy of LCD 3D printing technology. There is no study so far that compared DLP and LCD 3D printing technology. In 2014, Hazeveld et al. compared the accuracy of DLP 3D printing technology to PolyJet Photopolymer 3D printing technology. Their results suggested that the printer with PolyJet Photopolymer 3D printing technology is more accurate than the DLP 3D printer [34]. In 2018, Kim et al. compared SLA 3D printing technology, DLP 3D printing technology, Fused Filament Fabrication 3D printing technology, and PolyJet Photopolymer 3D printing technology [26]. They concluded that the precision of the PolyJet 3D printer was the best, followed by the DLP 3D printer and then the rest. The PolyJet 3D printer was the best in terms of trueness followed by the SLA 3D printer, DLP 3D printer, and last the Fused Filament Fabrication 3D printer. The same year, there were two different studies that evaluated the accuracy of 3D printing technologies by using PolyJet 3D printers and DLP 3D printers compared to dental stone models. The study of Brown et al. was the first study in that year, and they concluded that there was a statistically significant difference between the two printers and the dental casts but there was no clinically significant difference [30]. The second study was conducted by Park et al., and they concluded that the stone model is more reliable than the two printer models [29]. In 2020, Pereira et al. evaluated the difference in the accuracy between the DLP, Fused Filament Fabrication, and PolyJet Photopolymer 3D printers [27]. Their results suggested that the PolyJet Photopolymer 3D printer showed the best accuracy, followed by DLP. Lastly, in the same year, Akyalcin S. et al. compared DLP, SLA and PolyJet Photopolymer 3D printing technology [28]. They concluded that DLP and PolyJet Photopolymer 3D printers had the same accuracy in printing dental models. In January of 2022, Giudice et al. evaluated the accuracy of printing dental models by entry-level LCD 3D printers which they compared to an SLA 3D printer. They decided to evaluate the layer height as well, and for this reason, they printed the models in two different layer height settings (50 μm and 100 μm). Their results suggested that entry-level LCD 3D printers are not as accurate as SLA 3D printers in terms of trueness and precision. The layer height setting does not affect the accuracy of the prints. Lastly, they found that the entry-level LCD 3D printers will not affect the clinical results [37]. In the same year, and more specifically, in July of 2022, Venezia et al. compared the accuracy of four different 3D printing technologies. They used one DLP 3D printer, one entry-level LCD 3D printer, and two SLA 3D printers. Their results suggested that the LCD printer performed near the clinical threshold value by evaluating the trueness and precision of these technologies in printing models. They suggested that the entry-level LCD printer should be used cautiously for clear aligner production [38].

The results of the present study suggest that 3D printers based on DLP 3D printing technology can produce more accurate results than 3D printers based on LCD 3D printing technology in terms of printing dental models. In this study, we used the Flashforge Focus 9.25 6K, an LCD 3D printer, and the Flashforge Hunter, a DLP 3D printer. Flashforge Hunter performed the best in terms of trueness and precision. It is worth mentioning that even though the DLP 3D printer performed better in terms of accuracy, the accuracy results for both of these printers suggested that their prints will not negatively impact the orthodontic clinical outcome. The dental models are mainly used for orthodontic diagnosis and fabricating orthodontic appliances. More specifically, and since they are more sensitive in terms of accuracy, orthodontic appliances can be accurately fabricated as long as the machine error is equal to or below 0.25 mm. This is because the threshold value of clinical acceptance for generating orthodontic movement by using aligners, which is a very sensitive appliance to fabricate, is 0.25 mm. This was proved by previous studies that suggested that the maximum tooth movement per aligner ranged from 0.25 to 0.30 mm. Consequently, 3D printers must generate dental models with accuracy errors below this range to fabricate orthodontic aligners [34,38,39,40]. Taking this into account, the printed models from either DLP or LCD 3D printers will definitely result in the fabrication of an accurate appliance. Lastly, an important difference between these two printers is the size of the building plate. The building plate of the DLP 3D printer is much smaller than the LCD 3D printer. This results in less production of printed models by one print with the DLP 3D printer than with the LCD 3D printer.

## 5. Conclusions

The Direct Light Processing 3D printer is more accurate in terms of dental model printing than the Liquid Crystal Display 3D printer. However, both DLP and LCD printers can accurately be used to print dental models for the fabrication of orthodontic appliances. An orthodontic practice could benefit more by using a professional LCD printer since they can print more dental models in one print than with a DLP printer.

## Figures and Tables

**Table 1:** Mean RMS value and standard deviation (SD) by printer type.

	Printer		
	DLP	LCD	
	Mean (SD)	Mean (SD)	*p*-value
RMS (mm)	0.16 (0.02)	0.21 (0.04)	0.004

**Table 2:** Mean RMS value and standard deviation (SD) by printer type.

	Printer		
	DLP	LCD	
	Mean (SD)	Mean (SD)	*p*-value
RMS (mm)	0.12 (0.02)	0.21 (0.04)	0.011
